# Keystone Veterinary Medical Association

**Published:** 1897-12

**Authors:** W. L. Rhoads

**Affiliations:** Secretary


### SOCIETY PROCEEDINGS.

### KEYSTONE VETERINARY MEDICAL ASSOCIATION.

THE monthly meeting was called to order on November 9, at 8.30 P.M., by Vice-President H. P. Eves, Dr. Pearson having sent word that it would be impossible for him to be present early. Those present were: Drs. J. Cheston Morris, Otto G. Noack, Robt. Gladfelter, John B. Raynor, G. W. Shaw, F. S. Allen, H. P. Eves, W. Horace Hoskins, W. S. Kooker, Chas. Lintz, Jas. T. McAnulty, Leonard Pearson, Jas. B. Rayner, W. L. Rhoads, Thomas B. Rayner, C. J. Marshall, John W. Adams, A. F. Schrieber. Also Messrs. J. N. Megary, Lewis D. Horner, A. E. Cunningham, Bassett Kirby, P. K. Jones, John J. Repp, Wm. Hughes, J. E. Spindler. After the regular routine of business, Dr. Adams, the essayist of the evening, not being present, Dr. Kooker reported a case which he had diagnosed as azoturia. After giving the history and treatment, from which he had received but fair results, he said he wanted advice. Dr. Hoskins thought from its history and progress he would consider it a case of immobility. Dr. Eves spoke of having several cases similar in one locality, and felt it was due to some cerebral lesion, but could not tell just what, as the attacks were of short duration, relief coming invariably in the shape of death.

Drs. Pearson and Adams now came in, and upon President Pearson taking the chair he called upon Dr. Adams for his paper on "Opacities of the Crystalline Lens in the Domestic Animals." Dr. Adams dealt with his subject strictly from a practical standpoint. He first gave the names of the many opacities known more commonly as cataracts, then told of the symptoms and conditions by which each might be known. He then told of the treatment and possible chances of recovery. He then explained the different operations and the many appliances from which one might choose.

Dr. Pearson now read a paper on "Methods of Meat-inspection," wherein he told of the various methods of meat-inspection now in vogue throughout the civilized world, and he offered many valuable suggestions for the improvement of the inspection of Philadelphia's supply of meat and milk. During the general discussion which followed he suggested that the killing here be done in central abattoirs, or that all killing be done at a fixed time, so the inspectors may more thoroughly do the work assigned them. Dr. Schrieber, one of the present inspectors, told of the work they were doing. He explained the present method of inspection, and told how and why they were greatly handicapped by the present conditions. Dr. Hoskins said the people of New York owed much to the Woman's Health Protective Association of their city for the improved inspection which they now had, they having fought for good, thorough inspection for seven or eight years, while the surrounding towns are satisfied to take any and all meats dumped upon their market stalls. Drs. Noack and Kooker thought we should give special attention to market inspection. Dr. Adams thought we should first control the killing through central abattoirs, then look after the markets for tainted and otherwise unwholesome meat. Dr. Allen spoke of the delivery of milk in glass jars, saying the milk-man would fill the jar coming from a pest-house and give it to the next customer. Dr. Morris said this was not the proper method of serving in glass jars. His method was to have all milk bottled at the dairy and sealed with a label bearing date of milking and address of owner; it could then at all times be traced, and if these jars were properly sterilized when returned home disease could not be carried.

An exceedingly interesting report from Dr. E. Mayhew Michener on "Intussusception,"[1] two feet three inches in length, of small intestines of colt; operation and recovery. Dr. J. Cheston Morris now told of the post-mortem held upon Chief Utan, who had so lately died at the Philadelphia Zoo, he having an invagination of four inches. Dr. Hoskins, as chairman of committee appointed to confer with the Woman's Health Protective Association, now reported just what had been done, and gave notice of meeting to be held by the Woman's Health Protective Association at an early date, to which all veterinarians would be more than welcome. The hour being late, the meeting adjourned, although there were a number who wished to report cases.

W. L. RHOADS,
Secretary.

